# Can we explain machine learning-based prediction for rupture status assessments of intracranial aneurysms?

**DOI:** 10.1088/2057-1976/acb1b3

**Published:** 2023-03-10

**Authors:** N Mu, M Rezaeitaleshmahalleh, Z Lyu, M Wang, J Tang, C M Strother, J J Gemmete, A S Pandey, J Jiang

**Affiliations:** 1 Biomedical Engineering, Michigan Technological University, Houghton, MI, United States of America; 2 Department of Management Science and Statistics, The University of Texas at San Antonio, San Antonino, TX, United States of America; 3 Department of Health Administration and Policy, George Mason University, Fairfax, VA, United States of America; 4 Department of Radiology, University of Wisconsin, Madison, WI, United States of America; 5 Department of Radiology, University of Michigan, Ann Arbor, MI, United States of America; 6 Department of Neurosurgery, University of Michigan, Ann Arbor, MI, United States of America; 7 Center for Biocomputing and Digital Health, Health Research Institute and Institute of Computing and Cybernetics, Michigan Technological University, Houghton, MI, United States of America

**Keywords:** intracranial aneurysm, machine learning, computational fluid dynamics

## Abstract

Although applying machine learning (ML) algorithms to rupture status assessment of intracranial aneurysms (IA) has yielded promising results, the opaqueness of some ML methods has limited their clinical translation. We presented the first explainability comparison of six commonly used ML algorithms: multivariate logistic regression (LR), support vector machine (SVM), random forest (RF), extreme gradient boosting (XGBoost), multi-layer perceptron neural network (MLPNN), and Bayesian additive regression trees (BART). A total of 112 IAs with known rupture status were selected for this study. The ML-based classification used two anatomical features, nine hemodynamic parameters, and thirteen morphologic variables. We utilized permutation feature importance, local interpretable model-agnostic explanations (LIME), and SHapley Additive exPlanations (SHAP) algorithms to explain and analyze 6 Ml algorithms. All models performed comparably: LR area under the curve (AUC) was 0.71; SVM AUC was 0.76; RF AUC was 0.73; XGBoost AUC was 0.78; MLPNN AUC was 0.73; BART AUC was 0.73. Our interpretability analysis demonstrated consistent results across all the methods; i.e., the utility of the top 12 features was broadly consistent. Furthermore, contributions of 9 important features (aneurysm area, aneurysm location, aneurysm type, wall shear stress maximum during systole, ostium area, the size ratio between aneurysm width, (parent) vessel diameter, one standard deviation among time-averaged low shear area, and one standard deviation of temporally averaged low shear area less than 0.4 Pa) were nearly the same. This research suggested that ML classifiers can provide explainable predictions consistent with general domain knowledge concerning IA rupture. With the improved understanding of ML algorithms, clinicians’ trust in ML algorithms will be enhanced, accelerating their clinical translation.

## 1. Introduction

Management of asymptomatic unruptured intracranial aneurysms (IAs) is a clinical dilemma [1–6]. To date, research efforts have been devoted to the early identification of rupture-prone IAs, reducing morbidity and mortality due to hemorrhage, while sparing treatment of IAs at low risk. In recent years, multivariate machine-learning (ML) approaches for the characterization of IAs (e.g., rupture risk, stability) have been gaining momentum. Five recent ML studies [7–11] have shown an accuracy of 75%–86% in predicting IA rupture status.

To translate those ML approaches into clinical practice, we must overcome one common criticism: Those ML methods might have good classification performance but do not provide sufficient explanations. Traditionally, ML algorithms that yield high prediction accuracy intend to be more complex and thus more challenging to interpret, as shown in Supplemental figure 1. Consequently, there is a need for balancing predictive accuracy and model interpretability in the medical domain, whereas such a need is challenging due to the emergence of explainable ML algorithms studied here.

Towards this end, the primary objective of this study was twofold. **First**, we aimed to demonstrate that ML algorithms with various algorithmic complexity can be explained. The explainability of ML methods is critical from the regulatory standpoint and in gaining the trust of physicians and patients. Six selected ML algorithms include multivariate logistic regression (LR), multiple layer perceptron neural network (MLPNN), support vector machine (SVM), random forest (RF), extreme gradient boosting (XGBoost), and Bayesian additive regressions trees (BART) [12]. Of note, those six ML algorithms cover a spectrum of ML algorithms with varying accuracy, interpretability, and complexity (see Supplemental figure 1). A subset of LR, SVM, XGBoost, MLPNN, and RF have been previously used in [7–11]. **Second**, we investigated the interpretability of these algorithms with respect to domain knowledge. If ML algorithms’ explanations are consistent with domain knowledge, our finding leads to an increased trust in ML approaches by clinicians and patients.

## 2. Methods and materials

### 2.1. Source of data

From an internal database, patient-specific IA models were created from medical imaging data (DICOM images of 3D rotational angiographies) acquired from three sources: the University of Michigan, Changhai Hospital (Shanghai), and the Aneurisk open-source repository (http://ecm2.mathcs.emory.edu/aneuriskweb/index).

### 2.2. Participants

The inclusion criteria include: (1) sufficient data quality to establish CFD models, (2) IA size limited to between 4 and 25 millimeters, (3) no presence of closely-spaced second IA, and (4) IA is in the anterior circulation. In particular, some patients have two closely spaced IAs that are either in tandem (one proximal and the other distal) or adjacent (aneurysms opposite on one vessel) [13]. The presence of two closely spaced IAs induces more complex hemodynamics compared with single IAs; thus, we excluded those closely spaced IAs in this study. As this is a retrospective analysis of existing data, only angiographic data acquired post-rupture were available for ruptured IAs.

Our internal database contained 127 cases from different patients with known rupture status, and after using the above inclusion criteria, 112 cases were included. Specifically, we excluded 2 IAs greater than 25 mm and 9 IAs smaller than 4 mm. Also, two pairs of IAs (i.e., 4 IAs) were excluded because they are closely spaced IAs.

### 2.3. Sample size

112 (44 ruptured and 68 unruptured) cerebral aneurysms were identified, and all aneurysms were saccular aneurysms from the intracranial internal carotid artery [ICA; 38], middle cerebral artery [MCA; 52], or anterior cerebral artery [ACA; 22], respectively. IA’s rupture statuses were known and gathered from the medical record. Note that 112 aneurysms were from 111 patients. In other words, two aneurysms were collected from the same patients, and the other 110 were acquired from different patients.

### 2.4. Statistical Analysis and Methods

**Figure 1.**
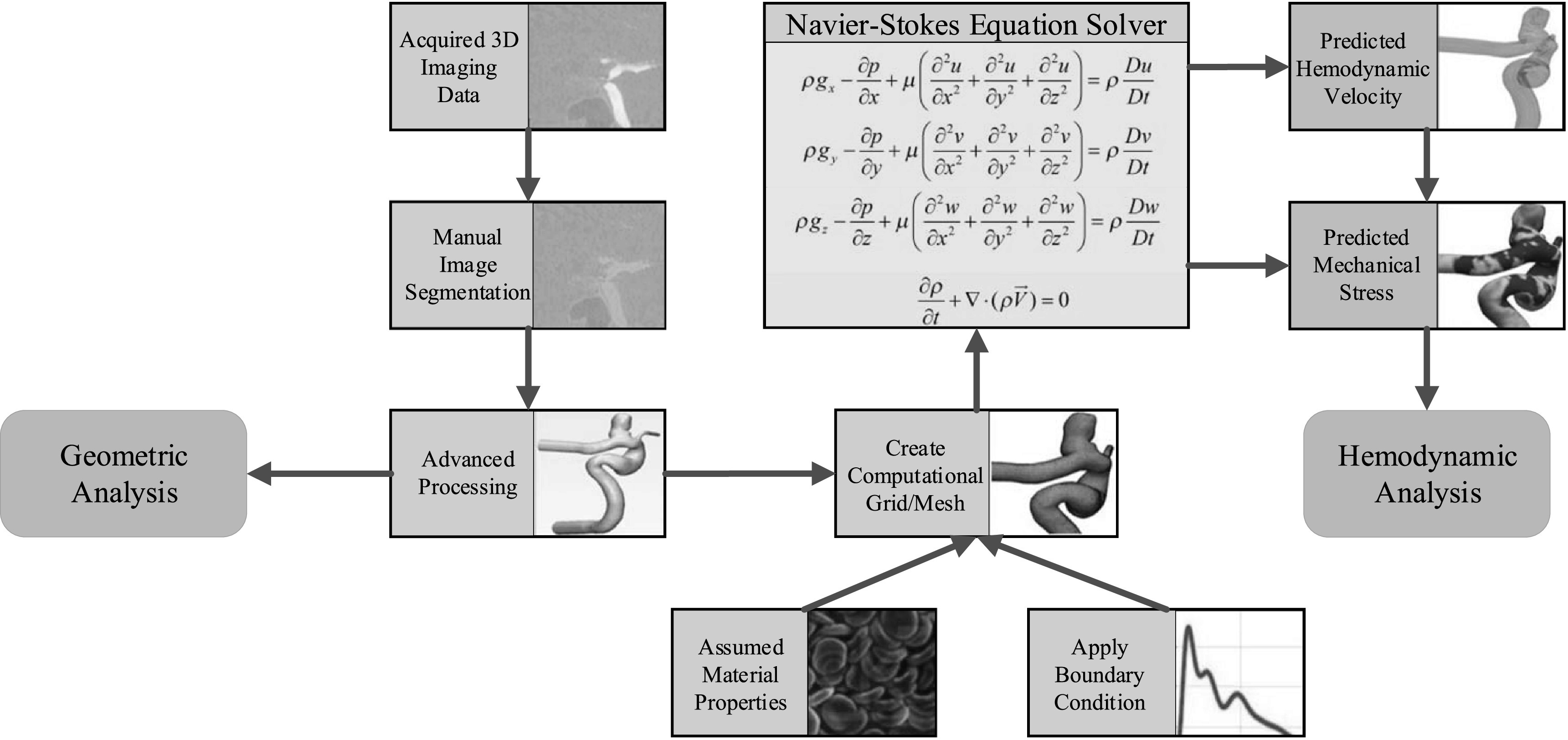
The overall workflow to acquire anatomical/hydrodynamic/morphological parameters.

The overall workflow of obtaining morphological and hydrodynamic parameters for subsequent ML-based prediction is shown in figure 1. Detailed protocols were previously published and can be found elsewhere [11, 13, 14] and were consistent with published protocols by others [15, 16]. The CFD workflow was verified with both phase-contrast magnetic resonance angiography (PC-MRA) [17, 18] and ultrasound Doppler [19].

More specifically, geometric analysis and CFD model creation were done by in-house Python scripts integrated into Vascular Modeling ToolKit (VMTK) [20]. A total of two anatomical features (Aneurysm Location and Type), nine hemodynamic parameters (Spatially and temporally averaged wall shear stress during peak systole [Systole STAWSS], wall shear stress minimum during peak systole [Systole WSSMin], wall shear stress maximum during peak systole [Systole WSSMax], spatially averaged oscillartory shear index [Mean OSI], one standard deviation of oscillartory shear index [Std OSI], time-averaged low shear area less than 0.4 Pa [TA LSA 2], one standard deviation of time-averaged low shear area less than 0.4 Pa [TA LSA Std 2], time-average degree of overlap between flow vortex cores during systole [Systole TADVO], and one standard deviation of time-average degree of overlap between flow vortex cores during systole [Systole DVOStd]), and thirteen morphological variables (Bulbous, Aneurysm Volume, Aneurysm Height, Sac Max Width, Size ratio between aneurysm height and parent vessel diameter [Size Ratio Height], size ratio between aneurysm width and parent vessel diameter [Size Ratio Width], Aspect Ratio Star, (parent) Vessel Diameter, Ostium Minimum, Ostium Maximum, Aneurysm (Surface) Area, Ostium Area, and Voronoi diagram characteristic curve points (V1 ∼ V11)) were calculated. For the sake of completeness, definitions of those parameters and their calculation methods are included in section B of Supplementary Materials.

The performance of a machine learning model is usually measured by bias (i.e., the difference between the actual value and the predicted value) and variance (i.e., the range and dispersion of the predicted value). Typically, for small training sets, a classifier with high bias/low variance (e.g., LR, Linear regression, Naive Bayes) is consistently more preferable than a classifier with low bias/high variance (e.g., SVM, Decision Tree, K-Nearest Neighbor) because the latter is prone to overfitting. However, as the training set grows, the classifiers with low bias/high variance will gradually show their advantages due to their lower asymptotic error. In contrast, a high-bias classifier is no longer sufficient to provide accurate predictions. In addition, compared with classifiers that are insensitive to missing data (e.g., XGBoost, Naive Bayes) [21, 22] and insensitive to noise and outliers (e.g., RF) [23, 24], some classifiers are greatly affected by the data quality, e.g., missing data (e.g., LR, SVM) [25, 26], noise (e.g., Decision Tree) [27, 28], sample imbalance (e.g., K-Nearest Neighbor) [29], and abnormal data (e.g., AdaBoost) [30, 31]. In this paper, six commonly-used ML algorithms (LR [25], SVM [32], RF [23], XGBoost [21], MLPNN [33], and BART [34]) were selected. A brief introduction of these 6 Ml algorithms and their pros and cons are provided in Supplemental Materials (Supplemental table 1). All ML algorithms were implemented in Python, and more details were provided in Supplemental Materials.

Once all geometric and hemodynamic data were obtained for each realization, the entire dataset was first randomly split into a training set (100 cases) and a testing set (12 cases) by a 9 to 1 ratio. Using the training dataset, optimal hyper-parameters for each model were auto-tuned during the training of 10-fold cross-validation, as the auto-parameter tuning is available in Scikit-learn. Since unruptured IAs count for the majority of cases in our database, which may bias the machine learning model, we set the parameter ‘class_weight’ of Scikit-learn classifiers to be ‘balanced,’ which can automatically calculate the weight and assign a higher weight to the ruptured class with fewer samples. In addition, we used the ‘GridSearchCV’ class to guide each classifier to automatically select the optimal training parameters, further ensuring the effectiveness of learning unbalanced data.

**Figure 2.**
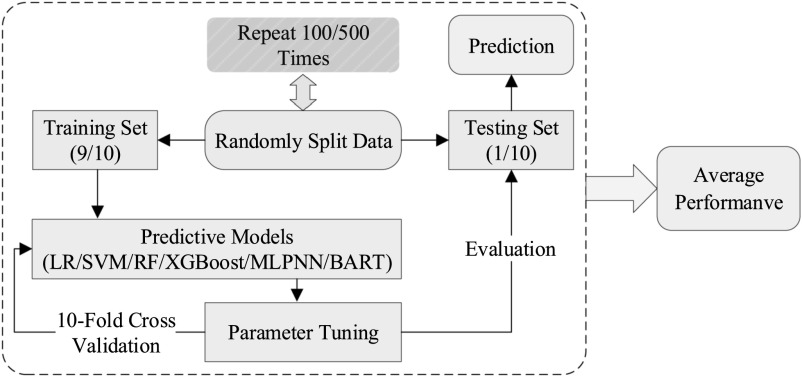
The training and evaluation processes for different machine learning models.

The ML models were finally tested on the testing dataset to conclude the analysis of each realization. This process was repeated 100 times to ensure that statistically stable results were achieved, as shown in figure 2.

The performances of 6 considered ML methods were assessed in terms of the prediction accuracy and area under the curve (AUC) of receiver operating characteristics on the testing samples. It is worth noting that the calculation of AUC considers the learner’s classification ability for both positive and negative examples, so it is not sensitive to whether the sample class is balanced and thus can make a reasonable evaluation of the classifier in the case of unbalanced samples.

The prediction accuracy score is calculated: ${\rm{Accuracy}}=(TP+TN)/(TP+FP+FN+TN),$where TP, TN, FP, and FN denote true-positive, true-negative, false-positive, and false-negative cases, respectively. Other evaluation metrics, including precision (also known as positive predictive value [PPV]), recall (also known as sensitivity), and F1-score (also known as a harmonic mean of precision and recall), were calculated via: ${\rm{Precision}}=TP/(TP+FP),$ ${\rm{Recall}}=TP/(TP+FN),$ and ${\rm{F1}}=2\times {\rm{Precision}}$ × ${\rm{Recall}}/\left({\rm{Precision}}+{\rm{Recall}}\right).$

Furthermore, in order to measure the authenticity of the prediction results, the standard deviation (SD) and confidence interval (CI) were measured. Generally speaking, SD measures the degree of variation between individual predictions, while CI can reflect the credibility of the overall prediction. Algorithmically, SD is calculated by $\sqrt{\displaystyle {\sum }_{i=1}^{N}{\left({x}_{i}-\mu \right)}^{2}/N},$ where $N$ denotes the number of predictions, ${x}_{i}$ denotes the result of each prediction, and $\mu $ denotes the average of all results. Based on the SD (denoted as $\sigma $), CI can be computed as $\left[\mu \pm {Z}_{\alpha /2}\times \sigma /\sqrt{N}\right],$ where ${Z}_{\alpha /2},$ and $\alpha $ denote the confidence coefficient and confidence level, respectively.

Global and local model-agnostic methods were adopted to evaluate aggregated and individual behaviors of each ML method, respectively. Global methods aim to describe how features affect predictions on average, while local methods explain individual predictions. For those global methods, it is possible to understand the model’s behavior as a whole, thereby revealing the influence of different elements of the model or input features on the prediction. However, the complexity of global interpretability is high, and for highly complex models, it is common to lower the bar for understanding an ML model to a modular level. Local methods attempt to explain how an ML model makes a specific prediction for a given instance/group of nearby instances. However, for a complex ML model, a set of simple rules can be used to describe the model locally, usually by partitioning samples into a neighborhood. As a result, predictions will be locally determined by only a small number of interactions from features with monotonic or linear effects. In general, the global and local methods do not conflict. A global understanding of an ML model can be achieved by aggregating local knowledge, and conversely, a single prediction can be explained by interpreting a model globally. In short, both global and local methods were used in this study to describe the overall and single predictions, aiding our comprehensive understanding of the intrinsic properties of different ML algorithms.

In terms of aggregated behavior or global interpretation, a permutation feature importance algorithm [35] was utilized to measure the importance of each feature. Loosely speaking, an importance score of a particular feature is obtained by computing an increment of the prediction error if the particular feature is removed during the permutation. Thus, a feature is considered important if the model error is increased if removed, as this observation indicates the ML model relies on that feature. The permutation feature importance algorithm was achieved by using the ‘PermutationImportance’ function from the ‘eli5’ package^8^ 
^8^

https://pypi.org/project/eli5
 of sklearn-compatible estimators on the Python Package Index (PyPI)^9^ 
^9^

https://pypi.org/
.

In order to investigate how each of the 6 Ml models reaches its decision, the local interpretable model-agnostic explanations (LIME) [36] algorithm was leveraged to train a local surrogate model (i.e., a simple linear model) to interpret individual predictions. Philosophically, the learned local surrogate model can be used to explain how a forecast is rendered. Algorithmically, LIME algorithm was implemented by importing the ‘LimeTabularExplainer’ function from the ‘lime’ package^10^ 
^10^

https://pypi.org/project/lime/
 on PyPI.

We also investigate how consistently the ML methods’ decision-making process can be reached between different methods. More specifically, using the best performer among six ML methods as a showcase example, we compared the feature (importance) ranking between the global permutation method [35] and SHapley Additive exPlanations (SHAP) algorithm [37]. Technically, the SHAP algorithm is mainly based on the ‘Explainer’ function of the ‘shap’ package^11^ 
^11^

https://pypi.org/project/shap/
. Furthermore, comparing LIME and SHAP algorithms cross-examined how features contribute to the final prediction for the best performer.

Section C of Supplementary Materials presents brief descriptions of the permutation feature importance algorithm, LIME algorithm, and SHAP algorithm.

## 3. Results

### 3.1. Model performance

The accuracy and AUC of each ML model were estimated, as summarized in table 1. The predictive accuracy of all ML models is comparable (74% to 81%), with XGBoost having the highest accuracy (81%) and the conventional LR yielding the lowest accuracy (74%). Regarding the AUC values, XGBoost also achieved the best performance (78%), with the LR being the lowest (74%). Results from the other three metrics (precision, recall, and F1) also showed six ML methods’ performance fell in a narrow range. Overall, XGBoost and LR were the best and worst performers, respectively. Following a similar process (see figure 2), results from splitting data into training and testing sets by an 8 to 2 ratio were nearly identical.

**Table 1.** Quantitative performance comparisons of six ML models in terms of different metrics. The 95% confidence intervals (CI) associated with each are provided in the shaded rows.

Metrics	LR	SVM	RF	XGBoost	MLPNN	BART
Accuracy/SD	0.74/0.11	0.77/0.11	0.76/0.10	0.81/0.08	0.76/0.11	0.77/0.10
95% CI	0.72–0.76	0.75–0.79	0.74–0.78	0.79–0.83	0.74–0.78	0.75–0.79
AUC/SD	0.71/0.12	0.76/0.12	0.73/0.11	0.78/0.09	0.73/0.11	0.73/0.11
95% CI	0.69–0.73	0.74–0.78	0.71–0.75	0.76–0.80	0.71–0.75	0.71–0.75
Precision (PPV)/SD	0.74/0.21	0.73/0.17	0.73/0.18	0.84/0.16	0.73/0.22	0.80/0.23
95% CI	0.70–0.78	0.70–0.76	0.69–0.79	0.81–0.87	0.69–0.77	0.76–0.84
Recall (Sensitivity)/SD	0.56/0.20	0.73/0.19	0.61/0.21	0.64/0.17	0.62/0.19	0.55/0.22
95% CI	0.52–0.60	0.69–0.77	0.57–0.65	0.61–0.67	0.58–0.66	0.51–0.59
F1 score (Harmonic Mean of PPV and Sensitivity)/SD	0.62/0.18	0.71/0.14	0.64/0.17	0.71/0.14	0.66/0.18	0.63/0.20
95% CI	0.59–0.65	0.68–0.74	0.61–0.67	0.68–0.74	0.63–0.69	0.59–0.67

Standard Deviation (SD), Confidence Interval (CI), Multi-variate Logistic regression (LR), Support vector machine (SVM), Random Forest (RF), Extreme gradient boosting (XGBoost), Multi-layer perceptron neural network (MLPNN), and Bayesian additive regression trees (BART).

The SD values and CI ranges in table 1 also indicate that the results of the six evaluation metrics for all of the six ML algorithms are reliable. We further used t-test^12^ 
^12^
According to the P value obtained by the t-test method, generally, P < 0.05 indicates a statistical difference, P < 0.01 means a significant statistical difference, and P < 0.001 is considered to have an extremely significant statistical difference. (calculated as $| {\mu }_{1}-{\mu }_{2}| /\sqrt{{\sigma }_{1}^{2}/{N}_{1}+{\sigma }_{2}^{2}/{N}_{2}},$ where $\mu ,$ ${\sigma }^{2},$ and $N$ denote the mean, variance, and the number of the sample, respectively) to measure the degree of difference between these results. In terms of accuracy, the results of the other five models have extremely significant or significant statistical differences from XGBoost (LR versus XGBoost, P = $6.63\times {10}^{-6};$ SVM versus XGBoost, P = 0.0115; RF versus XGBoost, P = 0.0001; MLPNN versus XGBoost, P = 0.0007; BART versus XGBoost, P = 0.0054). For AUC, except for the SVM model (SVM versus XGBoost, P = 0.4202), there are statistical differences between the results of other models and XGBoost (LR versus XGBoost, P = $1.89\times {10}^{-6};$ RF versus XGBoost, P = 0.0014; MLPNN versus XGBoost, P = 0.0053; BART versus XGBoost, P = 0.0021).

### 3.2. Model analysis

**Figure 3.**
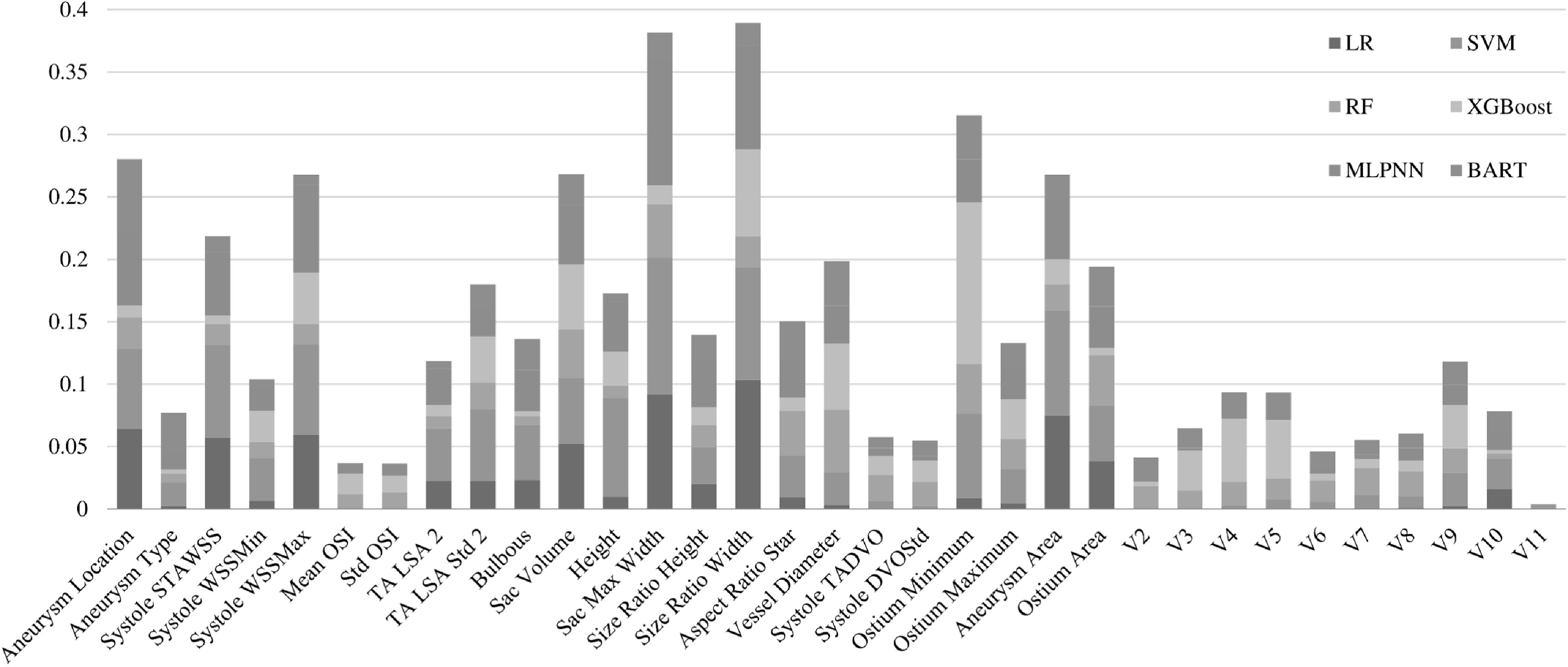
The importance of various features in predicting rupture status using six models.

We first used the permutation feature importance [35] algorithm to identify the top 12 features most critical to IA rupture predictions. Precisely, the importance scores of all six ML methods were summed together for a feature to obtain an ‘aggregated’ importance score for the feature, as shown in figure 3. We found that size ratio width is the most important feature that contributes to the identification of rupture status, followed by sac max width, ostium minimum, aneurysm location, and aneurysm volume in the top 5. The rest of the top 12 features include aneurysm area, systole WSSMax, systole STAWSS, vessel diameter, ostium area, TA LSA Std2, and aneurysm height.

Subsequently, the LIME [36] algorithm was used to analyze contributions from the top 12 features to understand how each ML method makes a prediction. Our findings are summarized in table 2. Based on the results of LIME, we divided the prediction rules of large or small chances leading to the ruptured or unruptured status of IAs into four categories: a larger value elevates the likelihood of having a ruptured status (see ‘HH’ in table 2); a larger value reduces the likelihood of having a ruptured status (see ‘HL’ in table 2); a smaller value increases the probability of having a ruptured status (see ‘LH’ in table 2); a smaller value reduces the probability of having a ruptured status (see ‘LL’ in table 2). In each of the four categories above, we quantified the predicted characteristics as ‘conform’ (see ‘√‘ in table 2) or ‘roughly conform’ (see ‘○‘ in table 2), where ‘conform’ indicates that all or almost all of our repeated 100 experiments (i.e., 90% or more) conformed to the predictive properties, and ‘roughly conform’ means that only the majority of the experiments (more than half) conformed to the corresponding rules.

**Table 2.** A summary of the influence of the top 12 features analyzed by LIME algorithm.

Parameters	LR				SVM				RF				XGBoost				MLPNN				BART			
	HH	HL	LH	LL	HH	HL	LH	LL	HH	HL	LH	LL	HH	HL	LH	LL	HH	HL	LH	LL	HH	HL	LH	LL
Aneurysm Area		√	√			○	○			○	√			○	√			○	√			√	√	
Aneurysm Location	√			√	√			√	√			√		√		○	√			√	√			√
Aneurysm Type	○			○	√			√	√			√	○			○	√			○	√			√
Systole STAWSS		√	√			√	√		√			√	○			√		√	√		√			√
Systole WSSMax	√			√	√			√	√			○	○			○	√			√	√			√
Aneurysm Volume	√			√	○		○			√	√			○	○		√			√		√	√	
Ostium Area		○	√			○	○			√	√			○	○			○	○			√	√	
Size Ratio Width	√			√	√			√	√			○	○			○	√			√	√			√
Vessel Diameter		○	○			○	√			√	√			√	√			○	○			√	√	
Systole DVOStd		○	○		○		√			√	√			○	○			○	○			√	√	
TA LSA Std 2		○	√			√	√			√	√			○	√			○	○			√	√	
Aneurysm Height	○		○		√			√	√			√	○		√		○			○		○		○

The √ indicates that the model conforms to the predicted characteristics, ○ indicates that the model roughly conforms to the predicted characteristics; a higher value leads to a higher chance of rupture (HH), a higher value leads to a lower chance of rupture (HL), a lower value leads to a higher chance of rupture (LH), a lower value leads to a lower chance of rupture (LL); Logistic regression (LR), Support vector machine (SVM), Random forest (RF), Extreme gradient boosting (XGBoost), Multi-layer perceptron (MLPNN), Bayesian additive regression trees (BART); Spatially-temporally averaged wall shear stress (STAWSS), Wall shear stress maximum (WSSMax), and Degree of volume overlap standard deviation (DVOStd).

**Figure 4.**
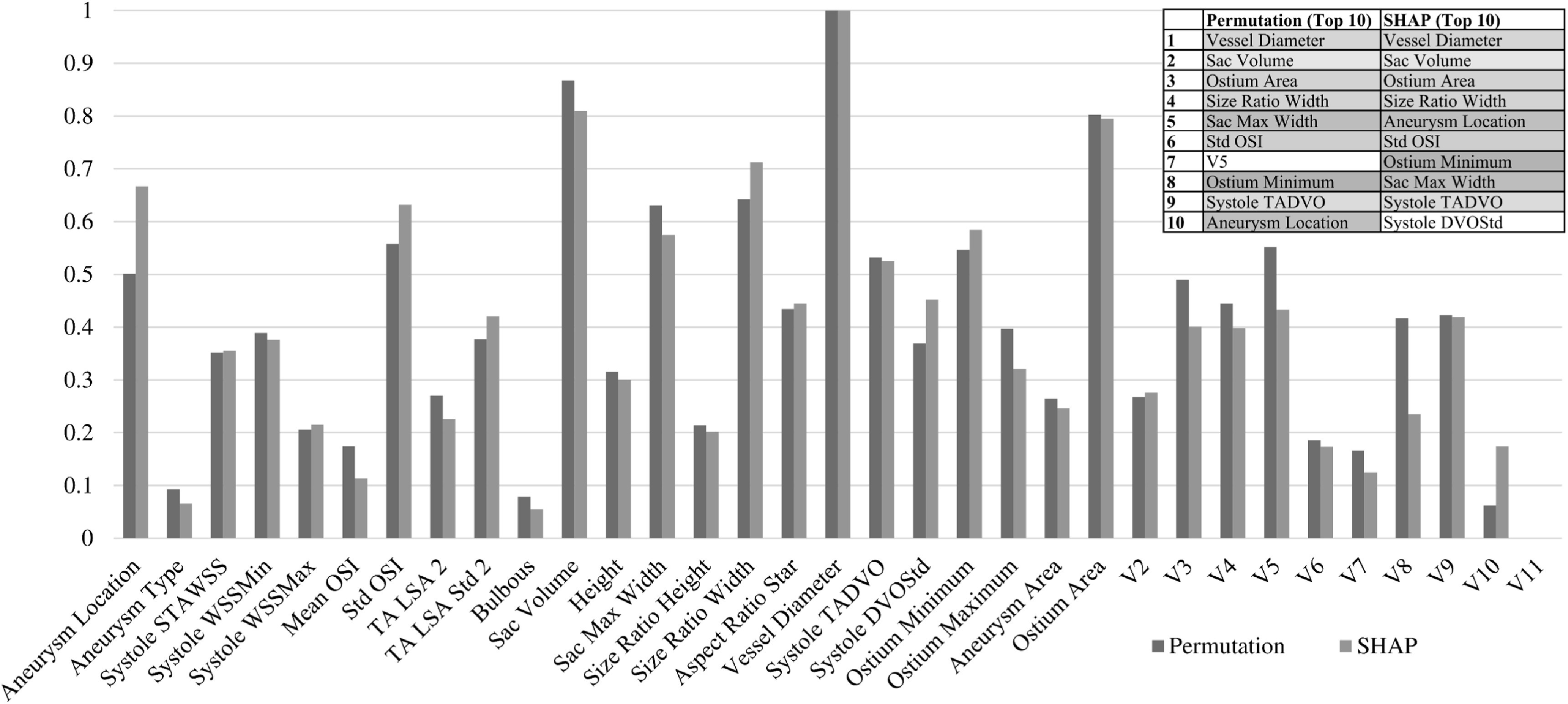
A comparison of feature importance measured from the XGBoost model using Permutation and SHAP algorithms. All importance metrics are normalized to [0, 1].

The XGBoost method has the best performance among the 6 Ml algorithms. Thus, we presented XGBoost model’s feature importance using both the permutation feature importance and SHAP algorithms as a showcase example. As illustrated in figure 4, both methods recognized that vessel diameter, sac volume, ostium area, and sac max width were the top four important features. Also, in general, 9 out of the 10 top features were the same even though their orders were different. Observations from the other 5 Ml methods were similar.

**Figure 5.**
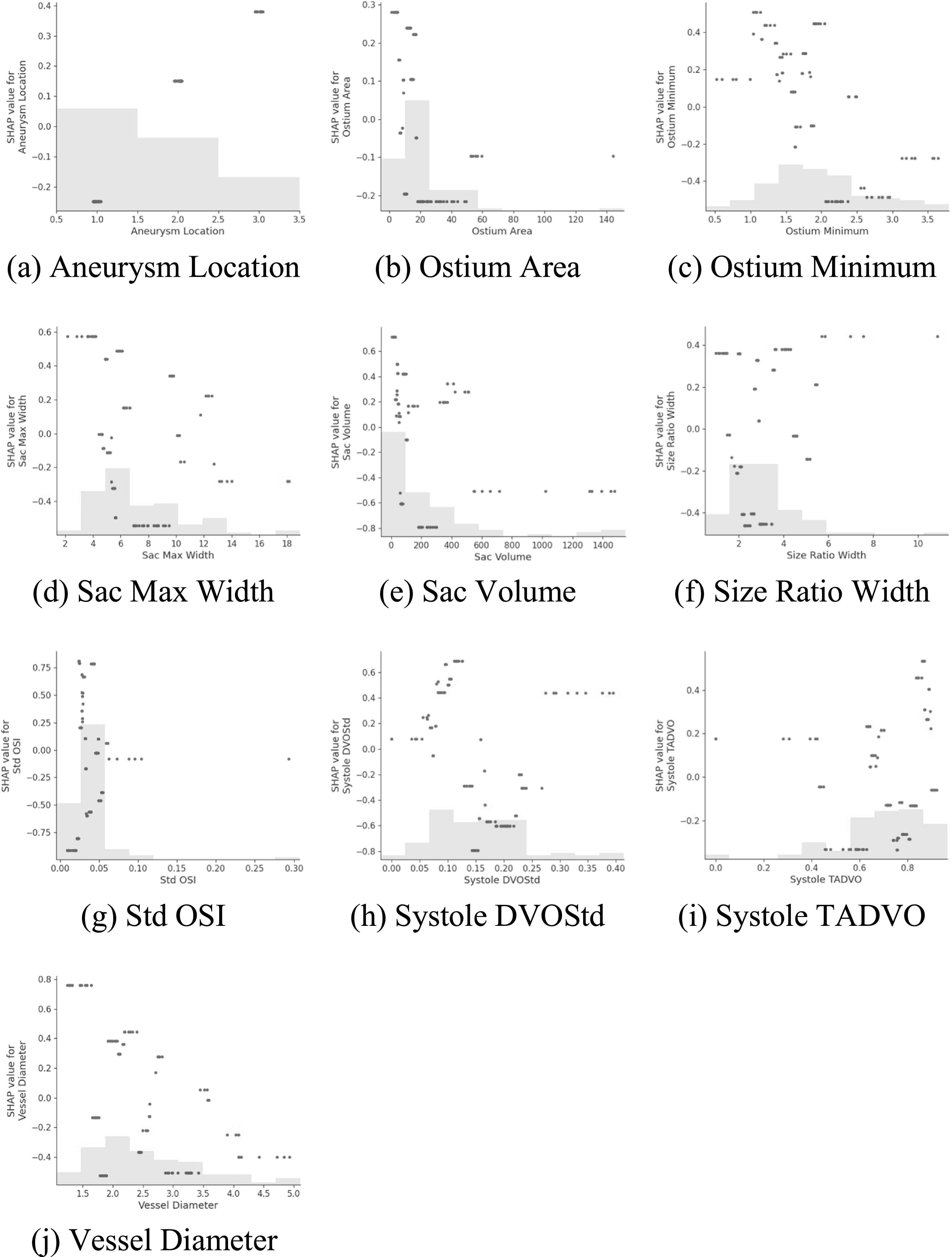
Dependency scatter plots showing the impact of a single feature on predictions by the XGBoost model. Each point indicates a single prediction from our data. The *x*-axis represents the value of the feature, and the *y*-axis shows the SHAP value of the corresponding feature (positive and negative values denote the ruptured and unruptured status, respectively), which means the influence degree of the feature value on the model prediction results. The light gray area at the bottom represents the histogram of the distribution in terms of data values.

We further leveraged the top 10 features obtained by the SHAP algorithm as a subgroup to perform XGBoost-based predictions. We also used the SHAP value to construct a dependency scatter plot to show the impact of individual features on model predictions. The dependency scatter plots of the top 10 features are shown in figure 5. We found that Aneurysm Location, Ostium Area, Ostium Minimum, Sac Volume, and Systole DVOStd are more instructive in predicting the rupture status of IAs.

In order to further analyze how consistently features can be explained, we used LIME and SHAP algorithms to interpret the prediction performance of XGBoost. In terms of how an individual feature is utilized for prediction, the overall trends obtained from LIME and SHAP algorithms are essentially the same, as summarized in table 3.

**Table 3.** Contribution of each feature during the XGBoost model’s prediction. Both LIME and SHAP algorithms were used to assess the contributions.

		LIME				SHAP			
Types	Parameters	HH	HL	LH	LL	HH	HL	LH	LL
Anatomical parameters	Aneurysm Location	√			√	√			√
	Aneurysm Type	√			√	√			√
Hemodynamic parameters	Systole STAWSS	√			√	√			√
	Systole WSSMin	√			√	√			√
	Systole WSSMax	√			√	√			√
	Mean OSI	○			√	○			√
	Std OSI		√		√		○		√
	TA LSA 2		○	√			√	√	
	TA LSA Std 2		√		√		√		○
	Systole TADVO	√			√	○			○
	Systole DVOStd		○	○			√	√	
Morphological parameters	Bulbous	√			√	√			√
	Aneurysm Volume		√	√			√	○	
	Aneurysm Height		√	√			√	√	
	Sac Max Width		√	√			○	√	
	Size Ratio Height	√			√	√			√
	Size Ratio Width	√		○		√		○	○
	Aspect Ratio Star		√		√	○	○		√
	Vessel Diameter		√	√			√	√	
	Ostium Minimum		√	√			√	√	
	Ostium Maximum		√	√			√	√	
	Aneurysm Area		○	√			√	√	
	Ostium Area		√	√			√	√	
	V1 ∼ V11		√	√			√	√	

The √ indicates that the model conforms to the predicted characteristics, ○ indicates that the model roughly conforms to the predicted characteristics; a higher value leads to a higher chance of rupture (HH), a higher value leads to a lower chance of rupture (HL), a lower value leads to a higher chance of rupture (LH), a lower value leads to a lower chance of rupture (LL); Extreme gradient boosting (XGBoost), Local interpretable model-agnostic explanations (LIME), SHapley Additive exPlanations (SHAP).

## 4. Discussion

Six ML methods showed comparable performance and similar interpretability patterns (table 2). The interpretability/explainability may be an essential requirement for an ML-based medical product. First, to improve the adoption of the proposed ML system, a black-box approach might be unacceptable for many physicians [38]. Second, regulatory bodies (European MDR and US FDA) or institutional review boards may request explainability when an ML-based product enters the clinical workflow.

In this sense, our study addressed a significant problem. To our knowledge, only one recent study [8] by Ou *et al* [8] investigated the relative importance of different features only for XGBoost using the SHAP analysis [37], whereas they did not consider hemodynamic parameters. In this study, we included 6 Ml algorithms using the LIME algorithm. However, different ML methods may select different features, as suggested by figure 3. If the 6 Ml methods were forced to use the same top 12 features, we found that the utility of the top twelve features by the 6 Ml methods was largely consistent. Particularly, how nine important features (Aneurysm Area, Location, Aneurysm Type, Systole WSSMax, Ostium Area, Size Ratio Width, Vessel Diameter, Systole DVOStd, and TA LSA Std2) contribute to the rupture status predictions was nearly the same in all 6 Ml algorithms. More evidence suggested that all investigated ML algorithms are explainable; thus, we may not consider those ML methods opaque. Traditionally, the best performer, XGBoost, is challenging to interpret (see Supplemental figure 1).

Furthermore, another contribution of this study was that we used both SHAP and LIME algorithms to interpret the XGBoost method; interpretations of the XGBoost results were essentially the same, improving our confidence in explainable ML analyses.

More specific observations from explainable ML analyses were summarized below in two subsections, followed by a discussion of limitations.

### 4.1. Model interpretation

#### 4.1.1. Comparison of reasoning behind six ML methods

Interestingly, all 6 Ml methods learned that the aneurysm (surface) area negatively correlates with rupture status when all other features remain unchanged. This observation implies that spherically-shaped IAs are less likely to rupture because their surface areas are the smallest, given the same aneurysm volume. Thus, what the 6 Ml algorithms learned was similar to that reported by Lindgren *et al* [39].

Results in table 2 related to aneurysm location indicate aneurysm location at the anterior cerebral artery (location value = 2) has an increased risk of rupture, while IAs located at the internal common carotid artery (location value = 1) have a lower risk of rupture. IA location is a recognized risk factor [40]; both prior research [15] and 5 out of 6 Ml models indicate that terminal IAs have an increased risk of rupture (table 2).

Regarding systole STAWSS, we observed split opinions (table 2). LR, SVM, and MLPNN found that systole STAWSS values inversely correlate with rupturing status/risk when all other features remain unchanged; RF, XGBoost, and BART believe otherwise. However, for systole WSSMax, we found that all six ML models learned that large and small WSSMax values correspond to elevated and reduced risks of IA rupture, respectively. It is important to recall that STAWSS is spatially and temporally averaged over the entire IA dome and, thus, is an index of overall flow activities with the IA dome. In contrast, a high WSSMax value is often a sign of concentrated flow jet(s) [41] within the IA dome. Thus, there is a consensus that high WSSMax values correlate to increased risks of IA rupture. The split view learned from 6 Ml algorithms perhaps is a reflection of the complex role of WSS during an IA’s pathological evolution, articulated well by Meng *et al* [42].

As far as aneurysm volume and aneurysm height are concerned, 6 Ml algorithms were divided again. Since using aneurysm size to predict IA rupture status yielded variable results in the literature, its contributions varied from model to model. Referring to the ostium area, all of the six ML algorithms believed that a higher ostium area value led to a reduced risk of rupture, while a smaller ostium area was associated with an increased chance of rupture. From hemodynamics, a small ostium area may be linked to flow jet formation or less flow entering the IA dome. That explains why an increased ostium area could lead to both elevated (jet formation) and reduced (reduction of flow entering IA) risks of IA rupture.

All 6 Ml models reached unanimous views regarding the size ratio width and (parent) vessel diameter. The size ratio is an aneurysm size normalized by its parent vessel diameter. The 6 Ml algorithm learned that a large size ratio corresponds to an increased risk of IA rupture, consistent with the clinical literature [43]. Now referring to the (parent) vessel diameter feature, a large (parent) vessel diameter effectively reduces the normalized aneurysm size and, therefore, correlates with a reduced risk of IA rupture.

In terms of the TA LSA Std 2 feature, all 6 Ml models suggested that lower and higher TA LSA Std 2 values have elevated and reduced risks of IA rupture, respectively. It is worth noting that high TA LSA Std 2 values often correspond to flow stagnation within an IA dome. Slow flow or flow stagnation promotes inflammation-related changes at the aneurysm wall that weaken its structural integrity [44].

The role of the systole DVOStd feature is a little different, as we learned from the 6 Ml algorithms. From table 2, all of the 6 Ml algorithms showed that lower systole DVOStd values lead to an elevated risk of IA rupture. That makes sense because, in principle, systole DVOStd reflects gross flow pattern changes within a cardiac cycle, with a high value indicating more dramatic changes over a cardiac cycle. In other words, a low DVOStd value suggests flow stagnation that promotes aneurysm wall inflammation, thereby leading to an elevated risk of IA rupture. However, the 6 Ml algorithms were divided on the elevated systole DVOStd values. When all other features remained unchanged, 5 out 6 Ml algorithms (LR, RF, XGBoost, MLPNN, and BART) believed that large systole DVOStd values correlate to a high risk of rupture, whereas SVM’s view was entirely different. This finding motivated us to further our understanding of vascular remodeling in the presence of significant gross aneurismal flow changes.

#### 4.1.2. Consistency during interpreting ML models

Using the best performer, the XGBoost model, as a showcase example, we found that multiple analytic methods result in nearly the same interpretations. Though their orders differed, the top 10 features ranked by their importance were the same by the permutation and SHAP methods (figure 4). Furthermore, we investigated how the XGBoost model utilized the top 12 features for making a prediction. We found that both SHAP and LIME algorithms give essentially the same results (table 3).

### 4.2. Limitations

Our study limitations include a small sample size and the retrospective nature of our analysis. Furthermore, in this study, the performance of ML methods was automatically tuned to avoid biases. However, such parameter tuning was limited to a narrow, practical range, based on our experience. More studies are needed to verify our findings. Another limitation of our study was that there are currently no available data enabling us to perform forward predicting of IA rupture risk for a fixed period.

We also recognized that three technologies (i.e., permutation, SHAP, and LIME algorithms) are not sufficient to serve as gold standards to decipher ML’s predictions fully. However, in our opinion, they do serve as a tool for performing valid explorations. For instance, by comparing the reasoning among six ML methods using LIME, we found that ML methods are in good agreement in terms of feature utility.

## 5. Conclusion

Our preliminary results suggested that explainable ML is feasible, though more work is needed to advance this line of translational research. Given the data investigated, we found that the XGBoost algorithm achieved the best overall performance and could be well explained using ‘explainable AI’ methods (LIME and SHAP). Overall, different ‘explained AI’ technologies gave essentially the same reasoning for the XGBoost algorithm’s predictions. We also found that ‘explainable AI’ methods could gain insight from the other 5 Ml-based predictive modeling methods.

Eventually, using available explainable ML technologies to augment clinicians’ understanding of ML’s rationales will bridge the above-said adoption gap, accelerating ML methods’ clinical adoption. The initial results reported here are encouraging and warrant further investigation. With the improved understanding of ML algorithms, clinicians’ trust in ML algorithms will be enhanced, accelerating their clinical translation. For future work, we aim to collect patients under imaging surveillance of their IAs so that their growth status is known and validated with ML algorithms.

## Data Availability

The data that support the findings of this study are openly available at http://github.com/jjiang-mtu/IA-rupture-prediction.

## Compliance with ethical standards

The authors declare no competing interests. The study is funded by a research grant from the National Institutes of Health (R01-EB029570A1). Nan Mu receives support from a Postdoctoral Fellowship Award from the American Heart Association (23POST1022454). This study involves a secondary analysis of existing data and has been approved by the institutional review boards at Michigan Technological University and the University of Michigan. No informed consent is needed.
